# Supplementary material for: Translating Virtual Reality Cue Exposure Therapy for Binge Eating into a Real-World Setting: An Uncontrolled Pilot Study
Source: J Clin Med. 2021 Apr 5;10(7):1511. doi: 10.3390/jcm10071511 (PMC8038593; doi:10.3390/jcm10071511)
Supplement: Supplementary file 1 [file jcm-10-01511-s001.zip › jcm-1150043-supplementary/Supplementary Materials/JCM_Supplementary_Table1.docx]

*Table S1: Food List*

1. Cake

2. Pizza

3. Potato Chips

4. Hot-dog

5. Cookies

6. Ice-cream

7. Chocolates

8. Bread

9. Donuts

10. Brownies

11. Pasta

12. Rice

13. Fruit

14. Salad

15. Yogurt

16. Waffles

17. French fries

18. Lean meat

19. Fried chicken

20. Peanut butter

21. Crackers

22. Popcorn

23. Pastries

24. Cereal

25. Bacon

26. Steak

27. Baked potato wedges

28. Hamburgers

29. Granola Bars

30. Lasagna
