# Supplementary material for: Translating Virtual Reality Cue Exposure Therapy for Binge Eating into a Real-World Setting: An Uncontrolled Pilot Study
Source: J Clin Med. 2021 Apr 5;10(7):1511. doi: 10.3390/jcm10071511 (PMC8038593; doi:10.3390/jcm10071511)
Supplement: Supplementary file 1 [file jcm-10-01511-s001.zip › jcm-1150043-supplementary/Supplementary Materials/JCM_Supplementary_Table2.docx]

*Table S2: Binge and Purge Behavior Outcomes*

**Intent-to-Treat**

| *Per week* | *OBE’s*  *(N=9)* | | | *SBE’s*  *(N=9)* | | | *Purging*  *(N=2)* | | |
| --- | --- | --- | --- | --- | --- | --- | --- | --- | --- |
| **Timepoint** | **Pre** | **Post** | **Follow-Up** | **Pre** | **Post** | **Follow-Up** | **Pre** | **Post** | **Follow-Up** |
| *Mean* | 3.80 | 0.60 | 1.22 | 3.00 | 1.22 | 2.40 | 1.00 | 0.25 | 0 |
| *SD* | 1.20 | 1.00 | 1.80 | 2.54 | 1.14 | 2.56 | 1.41 | 0.35 | 0 |

**Completers**

| *Per week* | *OBE’s*  *(N=11)* | | | *SBE’s*  *(N=11)* | | | *Purging*  *(N=2)* | | |
| --- | --- | --- | --- | --- | --- | --- | --- | --- | --- |
| **Timepoint** | **Pre** | **Post** | **Follow-Up** | **Pre** | **Post** | **Follow-Up** | **Pre** | **Post** | **Follow-Up** |
| *Mean* | 3.27 | 0.94 | 1.20 | 3.09 | 1.27 | 2.43 | 1.00 | 0.25 | 0 |
| *SD* | 1.56 | 1.34 | 1.66 | 2.30 | 1.06 | 2.40 | 1.41 | 0.35 | 0 |
