# Supplementary figures and images for: Translating Virtual Reality Cue Exposure Therapy for Binge Eating into a Real-World Setting: An Uncontrolled Pilot Study
Source: J Clin Med. 2021 Apr 5;10(7):1511. doi: 10.3390/jcm10071511 (PMC8038593; doi:10.3390/jcm10071511)

*Photo S1: Environments and Example Foods*


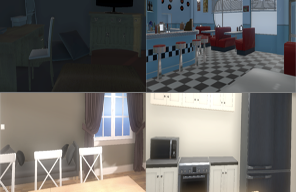


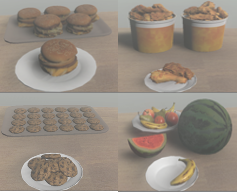

Supplement: Supplementary file 1 [file jcm-10-01511-s001.zip › jcm-1150043-supplementary/Supplementary Materials/JCM_Supplementary_Image1.docx]
